# Renal Involvement in Pediatric Small-Vessel Vasculitis: A Comprehensive Review of Clinical Impact, Diagnosis, and Management

**DOI:** 10.3390/medsci14020333

**Published:** 2026-06-20

**Authors:** Adina-Andreea Pop, Andreea Liana Bot (Rachisan), Emil Botan, Mihaela Sparchez

**Affiliations:** 1 Emergency Clinical Hospital for Children, 400023 Cluj-Napoca, Romania; 2 Department 2, Faculty of Nursing and Health Sciences, University of Medicine and Pharmacy “Iuliu Hatieganu”, 400023 Cluj-Napoca, Romania; 3 Pediatric Nephrology Department, 400023 Cluj-Napoca, Romania; 4 Department of Pathology, Cluj-Napoca County Clinical Emergency Hospital, 400006 Cluj-Napoca, Romania; 5 2nd Pediatric Discipline, Mother and Child Department, Faculty of Medicine, Iuliu Haţieganu University of Medicine and Pharmacy, 400023 Cluj-Napoca, Romania

**Keywords:** pediatric renal vasculitis, ANCA-associated vasculitis, IgA vasculitis, kidney involvement

## Abstract

**Background:** Renal vasculitis encompasses a heterogeneous spectrum of disorders where vascular inflammation leads to organ dysfunction. Given that renal involvement is the primary determinant of long-term morbidity, timely diagnosis and intervention are paramount. This review aims to synthesize recent pathogenic insights and evaluate how these mechanistic breakthroughs are reshaping current diagnostic and therapeutic paradigms. **Methods:** A narrative review of the literature was performed to analyze the pathophysiology, diagnosis, and management of pediatric renal vasculitis. The analysis synthesizes current clinical guidelines and recent trial data, highlighting the transition toward biomarker-driven precision medicine for refined disease assessment and management. **Results:** Diagnosis remains multimodal, necessitating the integration of clinical, laboratory, and histopathological data. In ANCA-associated vasculitis (AAV), recent evidence has challenged the traditional “pauci-immune” concept. Management of pediatric IgA vasculitis utilizes a risk-stratified approach, whereas cryoglobulinemic vasculitis requires targeted trigger elimination. Across all pediatric syndromes, there is a shift toward minimizing corticosteroid exposure and utilizing individualized frameworks. **Conclusions:** Despite substantial progress in targeted biological therapies and reduced corticosteroid burden, the long-term morbidity of pediatric renal vasculitis remains substantial. Outcomes are dictated by a synergy of disease-specific and patient-specific factors. Addressing persistent unmet needs in the field requires further refinement of individualized management protocols and the continued validation of dynamic biomarkers, alongside the implementation of pediatric-specific guidelines and age-appropriate outcome measures.

## 1. Introduction

Vasculitis comprises a heterogeneous spectrum of disorders characterized by immune-mediated inflammation of blood vessels, resulting in vascular structural damage and tissue ischemiai and subsequent organ dysfunction. The clinical presentation across this spectrum varies profoundly, ranging from relatively benign, self-limiting entities to life-threatening systemic phenotypes, such as Hughes–Stovin syndrome [1].

To address this heterogeneity, modern nomenclature and classification frameworks integrate vessel size, organ-specific predilection, and underlying immunopathogenic mechanisms to standardize management and trial design [2,3]. Despite significant advances in diagnostic and therapeutic strategies, pediatric vasculitis continues to impose a substantial morbidity burden. This is largely due to the involvement of vital organs. Specifically, kidney involvement represents the most clinically relevant prognostic factor, particularly in small-vessel vasculitis [4,5,6,7].

Among the broad spectrum of vasculitic syndromes, small-vessel vasculitis constitutes the predominant cause of primary glomerular disease. These disorders are traditionally divided into pauci-immune vasculitis and immune complex-mediated vasculitis (ICV), a categorization based on histopathological findings. Such distinct pathogenic pathways drive divergent histopathological and clinical patterns [2,8,9].

Renal vasculitis most commonly presents as glomerulonephritis, classically characterized by microscopic hematuria, proteinuria, and variable degrees of kidney dysfunction [3,10,11]. The presence and severity of kidney disease frequently dictate the requirement for aggressive immunosuppression [10,11].

Significant progress over the last decade has substantially refined the understanding of the immunopathogenesis of vasculitis. In ANCA-associated vasculitis (AAV), the alternative complement pathway has emerged as a central mediator of vascular inflammation [6,12,13]. These mechanistic insights have translated into targeted therapies, contributing to a paradigm shift toward steroid-sparing strategies [14,15,16]. Concurrently, diagnostic and prognostic accuracy have improved through the implementation of standardized scoring systems. Kidney biopsy remains the gold-standard diagnostic tool. Nevertheless, there is a growing emphasis on dynamic, biomarker-driven frameworks to enable precision medicine in vasculitis [8,17,18,19].

This narrative review summarizes current insights into vasculitis with renal involvement, with a specific focus on AAV, IgA vasculitis (IgAV), and cryoglobulinemic vasculitis (CV). We intend to synthesize recent mechanistic advances and their correlation with clinico-histologic phenotypes and targeted therapies. Additionally, we discuss how integrating novel biomarkers supports a shift toward precision medicine.

## 2. Classification

The 2012 Revised International Chapel Hill Consensus Conference (CHCC) nomenclature provides a standardized framework that stratifies vasculitis according to the predominant caliber of the involved vasculature, categorizing them as large-, medium-, and small-vessel diseases. While renal manifestations are the hallmark of small-vessel vasculitis, renal involvement also occurs in medium- and large-vessel vasculitis [2].

### 2.1. Small-Vessel Vasculitis

Small-vessel vasculitis targets the microvasculature, including arterioles, capillaries, and venules. This category is subdivided into ICV and pauci-immune vasculitis (typically ANCA-associated). AAV—comprising granulomatosis with polyangiitis (GPA), microscopic polyangiitis (MPA), and eosinophilic granulomatosis with polyangiitis (EGPA)—is histologically defined by pauci-immune necrotizing inflammation. Conversely, ICV—exemplified mostly by IgAV and CV—demonstrates intense immune complex deposition on immunofluorescence microscopy [2,20,21,22,23].

### 2.2. Medium-Vessel Vasculitis

Medium-vessel vasculitis, such as polyarteritis nodosa and Kawasaki disease, primarily affects the medium-sized, muscular arteries. Renal compromise typically arises from macrovascular ischemic injury, rather than intrinsic glomerular disease. Common renal manifestations are reduced blood flow, parenchymal infarction, and secondary hypertension [2,24,25].

### 2.3. Large-Vessel Vasculitis

Large-vessel vasculitis, including giant cell arteritis and Takayasu arteritis, seldom causes direct glomerular involvement. However, renal morbidity remains a concern, typically manifesting as renovascular hypertension and ischemic nephropathy [2,24,25].

## 3. Pathophysiology of Renal Vasculitis

Renal vasculitis arises from innate and adaptive immune-mediated inflammation of the endothelium. The kidney’s high perfusion and intricate capillary network render it uniquely susceptible to circulating autoantibodies and immune complexes, allowing even low-grade inflammation to cause significant kidney impairment [6,12,13,26]. Although both ANCA-associated and immune complex-mediated disorders frequently present with glomerulonephritis, the underlying pathogenic pathways remain markedly distinct.

### 3.1. ANCA-Associated Vasculitis: PR3 Versus MPO Phenotypes

AAV is the hallmark cause of pauci-immune necrotizing and crescentic glomerulonephritis. Emerging evidence suggests that PR3-ANCA and MPO-ANCA vasculitis are distinct disorders. PR3-ANCA disease is typically associated with specific variants in HLA-DP, SERPINA1, and PRTN3, as well as systemic inflammation and GPA. In contrast, MPO-ANCA vasculitis is linked to HLA-DQ polymorphisms and characteristically manifests as MPA [27,28,29].

At the level of the renal microvasculature, both PR3- and MPO-ANCA activate primed neutrophils by F(ab’)2 binding to surface-expressed PR3 or MPO antigens and to the neutrophil Fc-γ receptor via the Fc portion. This activation sequence triggers neutrophil-endothelial adhesion, followed by degranulation with the release of proteolytic enzymes (MPO, PR3) and complement factors, the production of reactive oxygen species, the synthesis and release of pro-inflammatory cytokines (IL-8), and NET formation [12,13,26,29].

### 3.2. Complement Activation and Endothelial Injury

Experimental data show that the C5a-C5aR1 axis is critical for ANCA-induced neutrophil activation and that a self-perpetuating cycle exists between ANCA-activated neutrophils and complement. These pathogenic insights have fundamentally reshaped the “pauci-immune” concept, demonstrating that AAV is a complement-amplified disease. Consequently, the role of complement in AAV has shifted from a passive bystander to a primary mediator of kidney injury, making it a critical therapeutic target. Randomized clinical trials (RCTs) have confirmed the efficacy of C5a receptor inhibition (Avacopan) [3,13,14,30].

### 3.3. Histological Scoring Systems in ANCA-Associated Glomerulonephritis

Renal biopsy in AAV remains indispensable for both diagnosis and prognostication. According to Berden’s histopathological classification, ANCA-associated glomerulonephritis is categorized into four classes: focal, crescentic, mixed, and sclerotic. This classification correlates strongly with renal outcomes; focal disease is associated with highly favorable recovery, whereas sclerotic lesions portend the poorest prognosis [3,8,31]. The ANCA Renal Risk Score (ARRS) and its updated iteration, the ANCA Kidney Risk Score (AKRiS), have further refined individualized risk stratification [31,32,33].

### 3.4. Immune Complex Vasculitis: IgA Vasculitis and Cryoglobulinemia

In contrast to pauci-immune diseases, ICV forms a distinct pathogenic category, characterized by prominent deposition of immunoglobulins and complement components within the glomeruli, triggering inflammation and kidney damage.

IgAV pathogenesis is driven by the production of galactose-deficient IgA1, which promotes the formation of anti-glycan IgG antibodies and circulating immune complexes. Their preferential deposition within the glomerular mesangium induces mesangial proliferation, cytokine release, and activation of the alternative and lectin complement pathways. Kidney involvement spans a spectrum from mild mesangial alterations to crescentic glomerulonephritis, with long-term prognosis closely linked to the severity of the initial histopathological lesions [9,21,34,35].

CV is defined by the presence of circulating immunoglobulins that precipitate at low temperatures. At the renal level, the deposition of cryoglobulins within glomerular capillary lumina induces complement activation and consumption, leading to endothelial damage. This injury typically results in a membranoproliferative glomerulonephritis (MPGN) pattern [2,9,22,36].

## 4. Clinical Manifestations of Renal Vasculitis in Children

The clinical spectrum of renal vasculitis ranges from indolent, asymptomatic urinary abnormalities to fulminant, rapidly progressive glomerulonephritis. The clinical presentation is determined by the underlying vasculitis subtype and its unique immunopathogenesis and is further modulated by patient-related factors such as age and comorbidities.

### 4.1. ANCA-Associated Renal Disease

The classic renal presentation of AAV is RPGN, characterized by microscopic hematuria, subnephrotic-range proteinuria, and a rapid decline in glomerular filtration rate (GFR), resulting in acute kidney injury (AKI). Constitutional symptoms, including fever, weight loss, and fatigue, alongside musculoskeletal findings such as arthralgia and myalgia, are common presenting features that reflect the multisystemic nature of AAV. However, the clinical phenotype in AAV is highly variable, correlating closely with the underlying serotype [3,27,28,37].

PR3-ANCA-positive vasculitis is most commonly associated with GPA, with kidney involvement reported in up to 83% of cases in pediatric series [5]. It typically presents with prominent extrarenal manifestations, particularly affecting the otolaryngologic region and the respiratory tract. The coexistence of systemic and renal features may facilitate earlier diagnosis [3,5,27,28].

In contrast, MPO-ANCA vasculitis more often presents with kidney-limited disease and fewer extrarenal manifestations in pediatric patients. Also, MPA tends to present at a younger age and may be more frequently associated with a more severe kidney disease compared with GPA in pediatric populations (primarily nephrotic-range proteinuria, requirement for dialysis, and end-stage renal disease) [5,7,28,29].

### 4.2. IgA Vasculitis and Cryoglobulinemic Renal Disease

As the most frequently diagnosed systemic vasculitis in pediatrics, immunoglobulin A vasculitis (IgAV, historically termed Henoch–Schönlein purpura) comprises the overwhelming majority of childhood vasculitis cases worldwide. The clinical trajectory for most affected children is characterized by an exceptionally favorable, self-resolving natural history. Approximately 94% of pediatric patients experience spontaneous complete remission of acute mucocutaneous and musculoskeletal manifestations, requiring minimal or no targeted therapeutic intervention [38,39].

Clinical manifestations of IgAV-associated nephritis (IgAVN) span a broad spectrum, ranging from isolated microscopic hematuria and mild proteinuria to more severe forms, including nephrotic syndrome and crescentic glomerulonephritis [21,35]. In the pediatric setting, kidney involvement typically occurs with a delay, emerging weeks after the first clinical presentation with the triad of purpura, arthralgia, and gastrointestinal manifestations. Nephritis occurs in 40–50% of children with IgAV and remains the primary determinant of long-term prognosis [6,21,34,40].

CV is exceedingly rare in children, and the literature consists primarily of case reports and small case series. In children, cryoglobulinemia is primarily driven by autoimmune diseases and essential cryoglobulinemia, followed by chronic infections (HBV, EBV, and CMV), primary immunodeficiencies, and, rarely, lymphoproliferative disorders [22,36].

CV typically manifests as a systemic small-vessel vasculitis, involving a combination of renal and extrarenal features. Patients typically present with proteinuria, microscopic hematuria, hypertension, and a distinct serological profile of profound hypocomplementemia, specifically involving C4. AKI frequently arises during disease flares and is often accompanied by systemic features such as palpable purpura, arthralgia, peripheral neuropathy, and fatigue [22,36].

### 4.3. Pediatric Versus Adult Renal Vasculitis

Although children with vasculitis often present with severe systemic inflammation, timely intervention optimizes renal recovery [4,5,7]. While adult patients are at a significantly elevated risk for end-stage kidney disease, the pediatric phenotype is more often self-limiting [21,34]. Consequently, distinct age-specific outcome measures, tailored therapeutic strategies, and age-adapted diagnostic vigilance are essential in clinical research.

## 5. Diagnostic Approach

The diagnostic workup for renal vasculitis is inherently multimodal, integrating clinical assessment and laboratory data with biopsy findings and, in selected cases, imaging studies.

### 5.1. Laboratory Investigations

Laboratory investigations serve as the initial diagnostic step in suspected renal vasculitis, providing critical insights into disease activity, immunopathogenetic profile, and the extent of multi-organ involvement. Antineutrophil cytoplasmic antibody (ANCA) serology is essential to the evaluation of small-vessel vasculitis, particularly when RPGN is suspected. Antigen-specific immunoassays for proteinase 3 (PR3) and myeloperoxidase (MPO) are indispensable for diagnosis, prognosis, and therapeutic guidance [3,10,19,28]. Historically, a two-tiered strategy was standard: initial screening via indirect immunofluorescence (IIF) on ethanol-fixed neutrophils to identify c-ANCA or p-ANCA patterns, followed by confirmatory antigen-specific PR3 and MPO immunoassays [41]. However, IIF is limited by subjective interpretation, inter-laboratory variability, and false-positive atypical patterns from infections or other autoimmune diseases; concurrent antinuclear antibodies (ANAs) can also obscure p-ANCA staining [42]. Following the Revised 2017 International Consensus Statement for GPA and MPA, the diagnostic paradigm shifted to bypass IIF in favor of primary screening via antigen-specific PR3 and MPO immunoassays. This strategy optimizes specificity, ensures reproducibility, and provides quantifiable titers that correlate with clinical phenotypes, prognosis, and induction therapy choices in pediatric small-vessel vasculitis.

Complement levels, particularly C3 and C4, enable clinicians to distinguish between pauci-immune and immune-complex-mediated vasculitis. Low serum complement levels, especially C4, strongly indicate immune complex-mediated etiologies, such as cryoglobulinemic or lupus-associated vasculitis [2,3,22]. Low serum C3 levels may also be present at diagnosis in AAV and correlate with more aggressive disease and worse renal prognosis [43,44]. To evaluate for secondary causes of renal vasculitis, autoimmune markers—including ANA, anti-double-stranded DNA antibodies, and serum cryoglobulins—are essential. While CRP and ESR are sensitive indicators of systemic inflammation, they lack disease specificity. Similarly, reduced GFR, hematuria, and proteinuria indicate glomerular injury but cannot differentiate specific vasculitic subtypes [3,10,19,22].

### 5.2. Kidney Biopsy

Kidney biopsy remains the gold standard for the diagnosis and prognostication of renal vasculitis [3,8,10]. On light microscopy, AAV is characterized by a necrotizing and crescentic glomerulonephritis. ICV often presents with mesangial or endocapillary hypercellularity, upon which necrotizing lesions and crescents may be superimposed. Cryoglobulinemia is notable for manifesting a membranoproliferative pattern, featuring distinctive intraluminal ‘pseudothrombi’ composed of precipitated cryoglobulins [2,8,10,21,22]. Immunofluorescence profiles offer further differentiation between these entities. ICV exhibits intense staining for specific immunoglobulins—dominant mesangial IgA1 in IgA vasculitis or capillary loop IgG/IgM in cryoglobulinemic vasculitis, alongside complement (C3). While AAV was traditionally defined by a ‘pauci-immune’ scarcity of such staining, high-sensitivity techniques, including immunohistochemistry and mass spectrometry-based proteomics, frequently detect C3d or C5b-9 within the glomeruli. These findings confirm localized activation of the alternative complement pathway [9,13,14,45]. For both entities, the proportion of reversible active lesions—cellular crescents and fibrinoid necrosis—against irreversible chronic damage—glomerulosclerosis and IFTA—is a key predictor of renal recovery following immunosuppressive therapy [8,32]. The recent advent of AI-assisted biopsy interpretation has revolutionized this assessment, enabling standardized, highly reproducible quantification [45,46].

### 5.3. Imaging Modalities

In the diagnostic workup of renal vasculitis, imaging plays a secondary, yet essential role, particularly in medium- and large-vessel vasculitis. In these cases, renal involvement is typically ischemic, requiring a macrovascular evaluation [2,24,25]. Doppler ultrasonography functions as a primary, noninvasive tool for assessing renal size, cortical thickness, and perfusion, simultaneously screening for obstructive causes of renal dysfunction [3,24,25].

High-resolution vascular imaging via computed tomography angiography (CTA) and magnetic resonance angiography (MRA) is essential for the management of medium- and large-vessel vasculitis. In conditions such as polyarteritis nodosa or Takayasu arteritis, these techniques permit the precise characterization of hallmark structural lesions, including arterial stenoses, aneurysms, and occlusions [24,25,36].

## 6. Management Strategies for Pediatric Renal Vasculitis

The management of renal vasculitis is centered on two primary objectives: achieving rapid disease remission and sustaining long-term quiescence. Therapeutic protocols are divided into induction and maintenance phases, with regimens tailored to vasculitis subtype, the severity of kidney involvement, histopathologic findings, and patient-specific factors [10,11,34,37].

### 6.1. Management of ANCA-Associated Vasculitis

#### 6.1.1. Induction Therapy

AAV typically presents as RPGN—a nephrologic emergency requiring immediate intervention to prevent irreversible renal failure. Recently published guidelines, including KDIGO 2024 [10], EULAR 2022 [47], CARRA 2022 [37], and ACR/Vasculitis Foundation 2021 [48], were developed primarily based on adult clinical trial data and also include pediatric recommendations. Induction therapy, as proposed by all these guidelines, combines high-dose glucocorticoids with either cyclophosphamide or rituximab [10,37,47,48]. High-dose intravenous methylprednisolone pulses remain the cornerstone of acute stabilization in severe ANCA-associated vasculitis (e.g., rapidly progressive glomerulonephritis or pulmonary–renal syndrome); however, maintenance strategies have shifted from prolonged high-dose oral corticosteroid tapers toward more rapid dose reduction [48]. This transition is underscored by the safety and non-inferiority results from the PEXIVAS and LoVAS trials [15,16].

Pivotal RCTs, including RAVE and RITUXVAS, have shown that rituximab is non-inferior to cyclophosphamide for inducing remission in ANCA-associated vasculitis [49,50]. Rituximab also demonstrated utility in patients with relapsing disease, PR3-ANCA positivity, or pre-existing contraindications to cyclophosphamide [24,49,50].

Cyclophosphamide has long served as the gold standard for inducing remission in AAV; however, growing concerns regarding cumulative toxicity—specifically infection, infertility, and malignancy—have catalyzed a shift toward rituximab as a preferred first-line alternative. Currently, cyclophosphamide is primarily reserved for life-threatening diseases or when rituximab is unavailable [3,10,37,49,50].

Current guidelines support the use of either agent and advocate a personalized induction strategy [10,47].

The role of plasma exchange (PLEX) in renal vasculitis has been substantially redefined. While previously regarded as a cornerstone therapy for severe ANCA-associated glomerulonephritis, the PEXIVAS trial did not demonstrate a clear long-term renal survival benefit compared with standard induction therapy [16]. As a result, current guidelines reserve PLEX for selected high-risk scenarios—such as severe acute kidney injury or life-threatening pulmonary hemorrhage [6,10,16,47].

Avacopan, a targeted C5aR1 inhibitor, is recommended as add-on therapy in adults with severe active ANCA-associated vasculitis and has received FDA approval based on the results of the ADVOCATE trial [14]. Pediatric data are lacking; however, its use aligns with the current therapeutic goal of minimizing glucocorticoid-related toxicity [14,15].

Methotrexate or mycophenolate mofetil may be considered as alternatives to rituximab for remission induction in non-organ- or non-life-threatening GPA or MPA [47].

#### 6.1.2. Maintenance Therapy

After remission induction, maintenance therapy is intended to preserve disease quiescence and prevent relapse. Evidence from the MAINRITSAN trial and RITAZ-AREM trial supports the preferential use of rituximab for maintenance, particularly in PR3-ANCA-positive disease. Both fixed-interval and biomarker-guided (CD19+ B-cell and/or ANCA) dosing regimens have proven highly effective [51,52]. Azathioprine, methotrexate, and mycophenolate mofetil stand as viable maintenance alternatives [2,10].

For both azathioprine and rituximab, the updated KDIGO guidelines recommend a maintenance duration of 18 months to 4 years following remission induction [10].

### 6.2. Management of Immune Complex Vasculitis

Management of immune complex-mediated renal vasculitis differs from that of AAV, necessitating a dual-therapeutic framework that targets both the underlying trigger and glomerular inflammation.

The IPNA 2025 and KDIGO 2025 guidelines represent the most current international guidance on the management of IgA vasculitis nephritis [11,34]. The most robust shared positions are as follows: (1) glucocorticoids should not be used prophylactically to prevent nephritis in IgAV; (2) renin–angiotensin system blockade (RASB) using ACE inhibitors or angiotensin receptor blockers (ACEi/ARB) is the cornerstone of proteinuria management once UPCR ≥ 0.2 mg/mg; (3) isolated hematuria without proteinuria does not warrant immunosuppression; and (4) cyclophosphamide should be restricted to rapidly progressive disease modeled on ANCA-AAV protocols [11,34].

IPNA pediatric recommendations provide a comprehensive management algorithm with explicit proteinuria-based treatment targets, graded biopsy indications, optional adjunct immunosuppressive agents, dosing protocols for children, and relapse management [34]. In alignment with the naturally benign, self-resolving evolution of the vast majority of pediatric presentations, the IPNA guidelines dictate that a kidney biopsy is not routinely indicated for mild phenotypes, with management centering exclusively on supportive care and optimized nephroprotection via renin–angiotensin system inhibition for persistent low-grade proteinuria. Conversely, aggressive immunosuppression is strictly reserved for the rare, severe cases; specifically, IPNA suggests treating children exhibiting nephrotic-range proteinuria or RPGN and histological risk for progression (ISKDC ≥ II) with a 3–6-month course of glucocorticoids, administered either as intravenous pulses followed by a tapering oral daily dose or as an exclusive oral regimen [34].

IPNA recommendations include therapeutic options such as calcineurin inhibitors (CNIs; tacrolimus, cyclosporine), mizoribine, and mycophenolate mofetil, either as steroid-sparing agents or in cases of severe disease (PCR > 2 mg/mg) and/or inadequate response to glucocorticoids [34].

According to IPNA, treatment of IgAVN in children should be maintained for at least 8–12 weeks, with immunosuppressive therapy discontinued only after ≥4 weeks of sustained remission—defined by normalization of proteinuria (PCR < 0.2 mg/mg), absence of gross hematuria, and normal renal function. The guideline also emphasizes structured long-term follow-up for at least 5 years [34].

Emerging therapies not yet included in current guidelines are being explored in pediatric and adult IgAVN. Telitacicept has shown promising results in a Chinese single-center study, with reductions in proteinuria and steroid-sparing effects [53].

Pediatric-specific data on CV nephritis are extremely limited, as cryoglobulinemia is rare in children and no pediatric-specific guidelines exist. Treatment recommendations are extrapolated from adult guidelines, having the general principles of (1) treating the underlying cause, (2) using immunosuppression for severe/organ-threatening disease, and (3) considering plasma exchange for life-threatening manifestations [22,36].

Rituximab and glucocorticoids are the key therapeutic agents and may be used in rapidly progressive or severe disease, while cyclophosphamide may be used as an alternative when rituximab is unavailable or ineffective [22,36].

Recent evidence suggests that eliminating the underlying trigger is a prerequisite for sustained remission. For example, in hepatitis C virus (HCV)-associated CV, the VASVALDIC trial demonstrated that direct-acting antivirals achieve clinical remission by eradicating the chronic viral stimulus [22,36,54].

Other potential therapies for refractory CV include mycophenolate mofetil and belimumab [55]. Belimumab combined with rituximab, a dual-stage B-cell-targeting strategy, represents a promising but investigational approach for non-infectious refractory CV [56,57].

Maintenance therapy adheres to the same individualized therapeutic principles, tailored to the patient’s etiology [22,36].

## 7. Prognosis and Outcomes

In systemic vasculitis, kidney involvement is a critical prognostic determinant, significantly impacting both patient survival and quality of life. The prognosis in renal vasculitis is determined by an interaction of disease-specific factors—such as subtype and histopathologic findings—and patient-specific variables, including age, comorbidities, and therapeutic response [7,8,32,33,58].

The clinical trajectory of AAV is strongly influenced by ANCA serotype. PR3-ANCA-positive disease is associated with higher relapse rates, whereas MPO-ANCA vasculitis typically presents with more advanced chronic kidney scarring [3,27,28,58].

Children with AAV frequently achieve high rates of inactive kidney disease, with remission reported in up to 83% at 12 months [4,7,30,59]. However, a substantial proportion (29–50%) progresses to end-stage kidney disease [5,30]. Renal function at diagnosis remains the strongest predictor of long-term outcome. Additional predictors of poor renal prognosis include sclerotic changes on kidney biopsy, hypertension at presentation, hypoalbuminemia, and failure to achieve remission following induction therapy [4,7,30,59].

Mortality rates are similar between pediatric and adult AAV (11.4% vs. 8.6%, *p* = 0.53) [60]. Due to higher relapse rates than in adults, children require longer maintenance therapy and closer monitoring [60].

In IgAVN, renal prognosis is heavily age-dependent. Pediatric patients generally achieve favorable outcomes and renal recovery, whereas adult-onset disease is often more aggressive, with kidney involvement being the primary driver of long-term outcomes. Progression to chronic kidney disease in IgAVN is strongly associated with persistent proteinuria, reduced eGFR at presentation, and crescentic lesions on kidney biopsy [10,34,35,61,62].

Available data indicate that 97% of pediatric cases develop renal involvement within six months of disease onset. Importantly, no long-term renal impairment has been reported in patients with persistently normal urinalysis. Consequently, even when initial urinalysis is normal, monitoring should be continued for six months; if findings remain normal throughout this period, further follow-up is not required [63].

CV is frequently marked by a relapsing-remitting course [64]. Kidney outcomes are influenced by the severity of glomerular injury, the burden of chronic histologic damage, and the effectiveness of treating the primary pathogenic stimulus [22,36,54,64].

Pediatric patients may have better outcomes if the underlying cause is identified and treated promptly, particularly given that children are less likely to have HCV-related disease with its associated complications (cirrhosis, lymphoma progression) [22,36].

Timely diagnosis and intervention are critical determinants of renal prognosis in vasculitic syndromes. Delays in recognizing renal involvement often lead to irreversible structural damage, which can preclude kidney recovery even when immunologic remission is achieved [3,24].

## 8. Precision Medicine, Relapse Prediction, and Unmet Needs in Renal Vasculitis

The management paradigm for renal vasculitis is undergoing a fundamental shift, driven by advances in immunopathology and molecular signatures, as well as therapeutic innovation. Standardized immunosuppressive protocols are increasingly being replaced by a personalized framework [3,10,11,28,33].

### 8.1. Toward Precision Medicine in Renal Vasculitis

Precision medicine in the renal involvement of small-vessel vasculitis is an emerging but not yet fully implemented field. It aims to improve diagnosis and to implement individualized therapy guided by molecular mechanisms, histopathologic phenotypes, validated biomarkers, and patient-specific safety profiles. While significant progress has been made in identifying biomarkers (particularly urinary sCD163 [17]) and stratifying patients by ANCA type, true precision medicine with biomarker-driven treatment algorithms remains aspirational. Pediatric-specific precision medicine data remain limited, though recent IPNA guidelines represent steps toward more individualized pediatric care.

Central to this paradigm shift is the distinction between PR3-ANCA and MPO-ANCA serotypes, which are now recognized as distinct disease entities with differing pathogenic mechanisms, relapse patterns, and long-term renal outcomes [27,28].

While CD19+ B-cell surveillance guides maintenance dosing, CD19-targeted CAR-T therapy represents an experimental, albeit promising approach for refractory ANCA vasculitis. It is hypothesized to induce a durable immunological reset and facilitate steroid-sparing remission [65,66]. Nevertheless, current clinical evidence is limited, and these findings remain hypothesis-generating.

### 8.2. Relapse Prediction and Biomarker-Guided Risk Stratification

Relapses remain one of the most challenging aspects in the longitudinal management of ANCA-associated vasculitis. Traditional relapse predictors, such as ANCA titers and clinical activity indices, exhibit suboptimal sensitivity and specificity when used in isolation.

While not yet integrated into routine clinical practice, risk stratification in renal vasculitis is advancing beyond standard markers (NGAL, KIM-1) toward molecular microenvironment mapping. Currently restricted to research, urinary “liquid biopsies” (sCD163, MCP-1, and CD4+ T cells) offer non-invasive tracking of glomerular activity distinct from systemic serology. Similarly, complement activation fragments represent promising, yet unvalidated, candidates for monitoring targeted therapies [17,18,19].

### 8.3. Clinical Scoring Systems and Renal Outcomes

Validated clinical indices, such as the Birmingham Vasculitis Activity Score (BVAS), standardize research and guide treatment in vasculitis but have limited long-term predictive power [3,10,32,33]. To bridge this gap, modern research increasingly relies on renal-specific outcome measures—such as eGFR trajectories, the magnitude of proteinuria reduction, and histological chronicity indices [3,10,33]. Relapse-prediction models are expected to evolve from ‘snapshot’ assessments toward integrating longitudinal biomarker trajectories with validated indices, such as BVAS, and objective renal outcomes [3,19,33].

### 8.4. Unmet Needs and Future Research Directions

Despite substantial progress, several unmet needs remain in the field of vasculitis. A major challenge is the lack of validated biomarkers to distinguish active inflammation from established chronic damage. Future studies should aim to define the “point of no return,” beyond which aggressive immunosuppression is unlikely to confer benefit [3,19,32,33]. Second, there is currently no composite framework that integrates clinical indices with histopathological and biomarker-derived data. Furthermore, a significant knowledge gap persists in pediatric vasculitis due to the scarcity of pediatric-specific data. The distinct clinico-immunological behavior of pediatric vasculitis underscores the urgent need for pediatric-specific biomarkers, outcome measures, and clinical trials. Finally, while emerging targeted therapies—such as complement inhibitors and CAR-T cell therapy—promise potent disease control with a more favorable toxicity profile, long-term safety and efficacy data remain sparse.

## 9. Conclusions

Across the spectrum of small-vessel vasculitis, kidney involvement remains a core determinant of both morbidity and long-term outcomes. Advances in immunopathogenic understanding have established that renal vasculitis encompasses biologically distinct diseases. The clear divergence in underlying mechanisms—spanning PR3-ANCA, MPO-ANCA, and ICV—has important diagnostic, therapeutic, and prognostic implications. Modern management guidelines promote steroid minimization strategies, in which targeted therapies (B-cell depletion, complement blockade) play an increasing role. Despite substantial therapeutic advancements, challenges remain in risk-stratifying relapses, distinguishing active vasculitis from established chronicity, and managing pediatric patients.

Contemporary practice is evolving toward a multidimensional framework that integrates dynamic biomarkers and longitudinal renal outcomes with standardized clinical indices. This comprehensive approach aims to enhance the assessment of disease activity, relapse risk, and therapeutic response. By leveraging collaborative, multicenter research incorporating histopathological and molecular insights, the field is moving beyond uniform treatment models toward truly individualized care.

## Data Availability

The original contributions presented in this study are included in the article. Further inquiries can be directed to the corresponding author.
